# Extraction, isolation, structural characterization, and antioxidant activity of polysaccharides from elderberry fruit

**DOI:** 10.3389/fnut.2022.947706

**Published:** 2022-07-19

**Authors:** Xinxin Wei, Junxiu Yao, Fangzhou Wang, Dejun Wu, Rentang Zhang

**Affiliations:** ^1^Key Laboratory of Food Processing Technology and Quality Control in Shandong Province, College of Food Science and Engineering, Shandong Agricultural University, Tai’an, China; ^2^Key Laboratory for Genetics and Breeding in Forest Trees of Shandong Province, Shandong Academy of Forestry Science, Jinan, China; ^3^Department of Food Science and Formulation, Gembloux Agro-Bio Tech, Université de Liège, Gembloux, Belgium

**Keywords:** elderberry, polysaccharide, separation and purification, structural characterization, antioxidant

## Abstract

The isolation, purification, and antioxidant activity of polysaccharides extracted from elderberry fruits were studied. Two neutral polysaccharides (EFP-0 and EFP-1) and three acidic polysaccharides (EFP-2, EFP-3, and EFP-4) were isolated from elderberry. EFP-0, EFP-1, EFP-2, EFP-3, and EFP-4 all contain arabinose, galactose, glucose, and mannose, with molecular weights of 1.7981 × 10^6^, 7.0523 × 10^6^, 7.7638 × 10^6^, 4.3855 × 10^5^, and 7.3173 × 10^5^ Da, respectively. Structural characterization showed that the backbone of EFP-2 consisted of →4)-Man*p* (1→4)-β-D-Glc*p* (1→ and →4)-β-D-Glc*p* (1→5)-α-L-Ara*f* (1→units, and T-α-L-Ara*f* (1→ and T-β-D-Gal*p* (1→ residues were detected by methylation analysis and NMR analysis. In addition, the MTT assay and zebrafish oxidative damage assay showed that EFP-2 had a protective effect on H_2_O_2_-damaged RAW264.7 cells in a dose-dependent manner, and zebrafish with the addition of EFP-2 would have low levels of ROS *in vivo* which showed significant antioxidant activity. Therefore, the results showed that the elderberry polysaccharides have antioxidant activity and can be used as potential antioxidants in functional foods.

## Introduction

The *Sambucus williamsii* Hance (elderberry), which belongs to the family Adoxaceae, is mainly distributed in Europe, Asia, and some parts of North Africa, and consists of seven different genera, divided into 5 to 30 species and 6 to 11 subspecies (1). Elderberry, black elderberry, European elderberry, and European black elderberry are common names for elderberry (2). Studies have shown that elderberry was both an edible and medicinal plant, which was the potential source of antioxidant ingredients (3) that can be used as a healthy dietary supplement in the preparation of jams and yogurt, and also applied in the treatment of common symptoms related to colds, fevers, coughs, and influenza (4). In addition, elderberry contains sugars, flavonoids, phenolic acids, lignans, triterpenoids, and other bioactive substances, so elderberries also have better physiological and pharmacological effects in antioxidants (5), anti-influenza (6), antiviral (7), anti-inflammatory (8), and anti-radiation (9).

Polysaccharides are a class of natural macromolecular biopolymers composed of multiple monosaccharides through glycosidic bonds (10). In recent years, plant, animal, and microbial polysaccharides have received widespread attention from the medical and food industries as bioactive ingredients and food additives. It is widely believed that polysaccharides extracted from natural products were less toxic and could be used as raw materials or supplements for functional food products (11), especially with antioxidants (12), anti-fatigue (13), anti-tumor (14), modulation of human immune function (15, 16), anti-inflammatory (17), hypolipidemic (18), and inhibition of cell proliferation (19), it can prevent the harmful effects of free radicals in the human body in terms of antioxidant (20, 21). Studies have shown that oxidative stress leads to a significant decrease in serum antioxidant activity and inhibition of superoxide dismutase, glutathione (GSH), total antioxidant activity, and glutathione peroxidase activities. Polysaccharides play a key role as edible free radical scavengers in inhibiting oxidative damage in organisms (22). Therefore, there was a need to study the structural characteristics of polysaccharides and use them as natural antioxidants to protect the body from excess reactive oxygen species invasion.

Polysaccharides are one of the important active substances of elderberry, with a total sugar content of 7.86∼11.50% and the reducing sugar content of 2.8∼8.55% in elderberry fruits (20). And elderberry fruits have a large yield, good color stability, and potential health-promoting effects, with wide applicability in different food applications, especially in products with long shelf life. Up to now, although polysaccharides extracted from various natural products had attracted significant research attention worldwide, little research has been reported on elderberry polysaccharides (23–25). Currently, only 10 saccharides or glycosides have been isolated from elderberry (26), and Liu et al. (27) and Wu (28) performed only preliminary extraction and purification of polysaccharides from Elderberry leaves and stems, and Song and Fu (29) concluded that Elderberry polysaccharides regulate insulin secretion from pancreatic islet cells and play a hypoglycemic role. The immunomodulatory effects of elderberry fruit polysaccharides were investigated by Lu et al. (30), who confirmed that elderberry polysaccharides stimulate immune responses in RAW 264.7 cells through the NF-kB pathway by the activity of *Escherichia coli* lipopolysaccharides, and similarly Stich et al. (31) who also demonstrated that polysaccharides in aqueous-derived elderberry extracts induce effective immunomodulatory effects. However, the isolation, purification, and structural characterization of elderberry polysaccharides have not been investigated. In this study, we isolated and purified elderberry polysaccharides from elderberry fruit residues and investigated their structural characteristics and antioxidant activity to initially elucidate the relationship between their structure and antioxidant activity.

## Materials and methods

### Materials and reagents

Elderberry fruits were harvested from the Qingzhou Elderberry-nursery base in Shandong Province, China, in August 2019, and the dark purple kernels growing in clusters were obtained (Shandong, China). DEAE cellulose 52 and Sephadex G-100 were provided by the Shanghai Yuanye Biotechnology Co (Shanghai, China). The monosaccharide standard methylation kit was provided by the Borealis Biotechnology Co., Ltd (Jiangsu, China). NaCl and trifluoroacetic acid (TFA) was provided by ACROS (Belgium). ROS fluorescent staining reagent CM-H2DCFDA was provided by the Beijing Biolab Biotechnology Co. Glutathione (GSH) was provided by Wuhan Rongcan Biotechnology Co., Ltd (Wuhan, China).

### Preparation, isolation, and purification of elderberry polysaccharides

#### Preparation of crude polysaccharides from elderberry

The protocol of hot water extraction of polysaccharides was according to the previous one with some modifications (32). The extract was refluxed twice with hot water at a material-to-liquid ratio of 1:20 (*w/v*) for 45 min each time, and the clarified solution was combined and extracted by filtration with a cloth funnel with diatomaceous earth while hot, concentrated at 65°C, alcoholic precipitation in 95% ethanol solution, followed by centrifugation at 10,000 × *g* for 10 min and freeze-dried to collect the precipitate. Then, the obtained polysaccharides were defatted and deproteinated by petroleum ether and Sevage reagent (chloroform: n-butanol volume ratio = 4:1). After that, the polysaccharide was alcoholically precipitated in 95% ethanol, centrifuged after 12 h in the refrigerator at 4°C, and the precipitate was used for freeze-drying to obtain the defatted and deproteinized crude polysaccharide of elderberry, which was EFP.

#### Isolation and purification of elderberry polysaccharides

**FIGURE 1:**
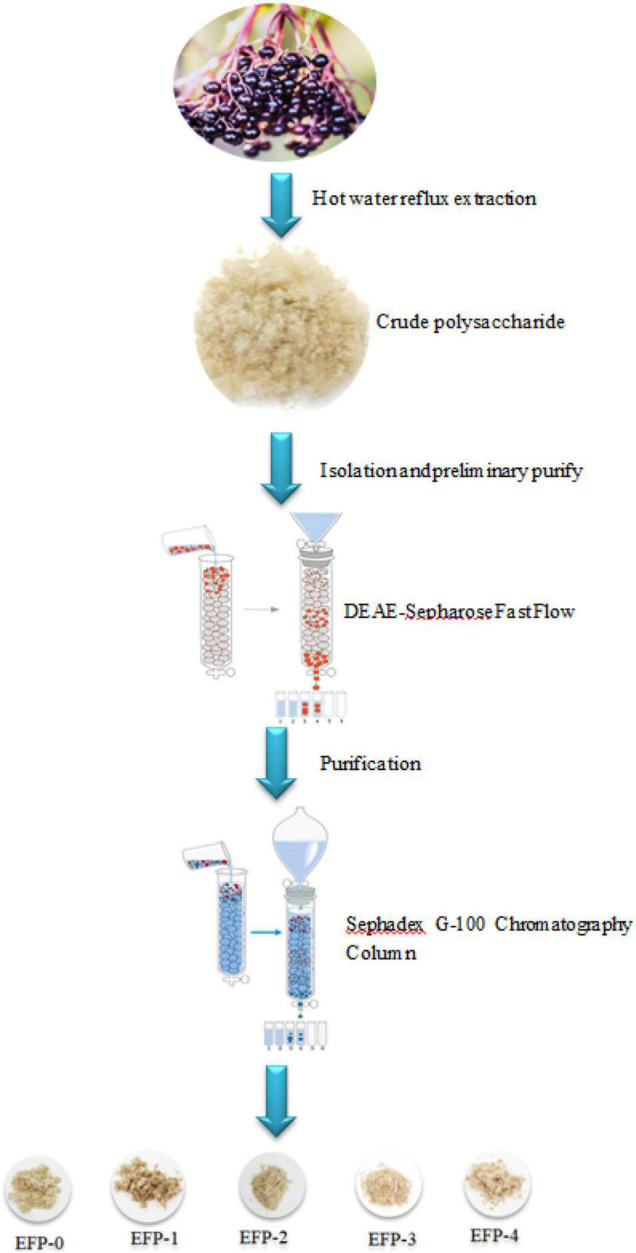
Flow chart of elderberry polysaccharide extraction, separation, and purification.

The polysaccharide separation and purification process were as shown in Figure 1. About 5.00 *g* of EFP was dissolved thoroughly in 100 mL of deionized water, followed by centrifugation at 10,000 × *g* for 10 min, and filtered through a 0.45-μm microporous filter membrane. The filtered crude polysaccharide solution was slowly added to a pre-equilibrated DEAE-cellulose 52 anion-exchange column (50 mm × 1,000 mm) and eluted with deionized water and 0.1, 0.2, 0.3, 0.4, and 0.5 mol/L NaCl solution, respectively, (33). Each tube was 50 mL with a flow rate of 5 mL/min, and 50 tubes of each were collected. The polysaccharide content of each tube was detected at 490 nm using the phenol-sulfuric acid method according to Ji et al. (33), and the elution curve was plotted with the number of tubes as the horizontal coordinate and the absorbance value as the vertical coordinate, and the polysaccharide eluates corresponding to the peaks (EFP-0, EFP-1, EFP-2, EFP-3, and EFP-4) were combined according to the peaks of the elution curve and spun off for concentration. 100 mL EFP-0 solution (0.5 mg/mL) was slowly added to Sephadex G-100 Sephadex column (16 mm × 2000 mm), eluted with deionized water, and 30 tubes of polysaccharide solution were collected at 10 mL per tube. The collected polysaccharides were lyophilized. EFP-1, EFP-2, EFP-3, and EFP-4 were purified by the above method.

#### Molecular weight determination

As previously described and improved upon Olawuyi et al. (34), the heavy mean molecular weight (Mw) distribution and polydispersity (Mw/Mn) of the five polysaccharide fractions were determined by high-performance gel permeation chromatography (HPGPC; 35). The samples and standards were weighed precisely, and the samples were prepared into a 5-mg/ml solution, centrifuged at 12,000 rpm for 10 min, then the supernatant was filtered through a 0.22-μm microporous membrane and the samples were transferred into a 1.8-mL injection vial. After that, 20 μL of sample solution was injected into a BRT105-104-102 series gel column (8 × 300 mm) with 0.05 mol/L NaCl solution as the mobile phase and the column temperature was 40°C at a flow rate of 0.6 mL/min. Using the detector RI-10A, the molecular weight size of each sample was calculated based on the standard curve.

#### Analysis of monosaccharide composition

The monosaccharide composition of purified polysaccharides was determined by ion chromatography (Thermo Fisher ICS5000, United States; 36). About 10 mg of lyophilized polysaccharide powder was placed in an ampoule, 10 mL of 3 mol/L TFA was added and hydrolyzed at 120°C for 3 h, and then the residual TFA was removed by rotary evaporator. A 10-mL acid hydrolyzed solution was accurately aspirated and blown dry in a tube, 10 mL of deionized water was added with vortex mixing, 100 μL of deionized water was aspirated, and 900 μL of deionized water was added and then centrifuged at 12,000 rpm for 5 min. The analysis was performed on a Dionex CarbopacTMPA20 (3 × 150) column with H_2_O and 15 mol/L NaOH and 100 mol/L NaOAC as mobile phases at a flow rate of 0.3 mL/min, column temperature of 30°C, and injection volume of 5 μL. The retention times and peak areas of the 16 monosaccharide standards were compared, and the different sugars were identified and quantified separately.

### Structure identification of elderberry polysaccharide

#### Infrared spectral analysis

The infrared spectral analysis was conducted following the methods described and improved by previous authors (20). The extracted and isolated purified elderberry polysaccharide was pressed into thin slices, and the slices were subjected to Thermo Nicolet IS10 Fourier transform infrared spectroscopy (FT-IR) with the setting of mid-infrared mode scan range of 4,000 to 400 cm^–1^, and the analysis was performed using OMSNIC 8.2 (Thermo Scientific™, United States) software.

#### X-ray diffraction spectrum

The maximum purity of polysaccharides in EFP-2 was obtained by isolation and purification, so the EFP-2 fraction was selected to investigate the structure and antioxidant activity of polysaccharides. As previously stated and modified (37), an X’Pert Pro X-ray diffractometer (PANalytical, Netherlands) was used to measure the crystallization properties of EFP-2 under 40 kV and 40 mA of radiation at Cu Kα (λ = 0.154 06 nm) on a copper target. The X-ray intensity was measured using a NaI crystal scintillation counter (scintillation counter) with a scanning range of 5° to 60°, a step size of 0.02°, and a scanning speed of 4°/min.

#### Scanning electron microscope

The study of the microstructure of elderberry polysaccharides was conducted by scanning electron microscopy (38). About 5 mg of the dried sample was taken, adhered to a conductive carbon film containing a double-sided adhesive, placed in the sample chamber of the ion sputterer, and sprayed with gold for about 40 s. After the sample was removed, it was placed in the scanning electron microscope observation chamber with an acceleration voltage of 2 kV for observation.

#### Methylation analysis

The methylation steps were mainly referred to Yang et al. (39) with some modifications. About 2 to 3 mg of EFP-2 sample was added to 1 mL of anhydrous DMSO, then the anhydrous base solution and iodomethane solution are quickly added and reacted for 60 min at 30°C in a magnetic stirring water bath, and finally 2 mL of ultrapure water was added to terminate the methylation reaction. The methylated polysaccharide was taken and hydrolyzed by adding 1 mL of 2 M TFA for 90 min and evaporated dry by a rotary evaporator. About 2 mL of double-distilled water and 60 mg of sodium borohydride have been added to the residue to reduce for 8 h, then, glacial acetic acid was added to neutralize the residue, 1 mL acetic anhydride was added to acetylate the reaction at 100°C for 1 h, and cooled. After that, 3 mL of toluene was added to remove the excess acetic anhydride. The acetylated product was dissolved with 3 mL of CH_2_Cl_2_ and the upper aqueous layer was removed. After, the CH_2_Cl_2_ layer was dried with an appropriate amount of anhydrous sodium sulfate, and the volume was fixed at 10 mL and put into a liquid phase vial. Finally, an RXI-5 SIL MS column of 30 m × 0.25 mm × 0.25 μm was used, the starting temperature was 120°C, ramped up to 250°C/min at 3°C/min, and held for 5 min. The injection port temperature was 250°C, the detector temperature was 250°C/min, and the carrier gas was helium at a flow rate of 1°mL/min.

#### Nuclear magnetic resonance spectroscopy

About 40 mg of EFP-2 was dissolved in D_2_O and then loaded in an NMR tube. The NMR spectra were scanned on a high-resolution 700 MHz Bruker AVANCE III NMR spectrometer (Bruker, Germany). One-dimensional NMR spectra (^1^H and ^13^C) and two-dimensional NMR spectra [correlation spectroscopy (COSY), heteronuclear single quantum coherence (HSQC), and heteronuclear multiple bond coherence (HMBC)] were adopted to analyze the structural features of polysaccharides (40).

### Effect of polysaccharides on the antioxidant activity of H_2_O_2_-induced oxidative damage in RAW264.7 cells

#### Cell culture

RAW 264.7 cell line was obtained from the Institute of Cell Biology, Chinese Academy of Sciences (Shanghai, China) and cultured in DMEM medium (with 10% fetal bovine serum and 100 U/ml each of penicillin and streptomycin) at 37°C, 5% CO_2_, and saturated humidity in an incubator (41).

#### MTT test

The viability of RAW 264.7 cells was determined by a standard assay using the MTT method (42). Cells of logarithmic growth phase were collected, washed with PBS buffer, trypsin digested, and centrifuged after the termination of digestion, and the cells were made into cell suspension with culture medium, inoculated into 96-well plates at a density of 5 × 10^4^ cells/well, and cell dilution was added at 100 μL/well and incubated for 24 h at saturated humidity, 37°C, and 5% CO_2_. The samples were diluted with culture medium separately, and 100 μL was added to each well; the solvent model group and control group (EFP-2 solution with different solubility) were also set up, and the final concentration of 1.2 mM H_2_O_2_ was added, respectively. After 4 h incubation, MTT solution was taken and added to the cells at 10 μL/well, and incubation was continued in the incubator for 4 h. After 4 h, the liquid in the wells was discarded, and 150 μL DMSO was added to each well to dissolve it. The absorbance at 490 nm was measured with an enzyme marker, and the survival rate was calculated according to the following Eq. (1).


(1) $$\mathrm{Survival}\mathrm{rate}\left(100\%\right)=1-\frac{\begin{array}{c} 100\%\mathrm{Absorbance}\mathrm{value}\mathrm{of}\mathrm{control}\mathrm{group}-\mathrm{Compound}\mathrm{absorbance}\mathrm{value} \end{array}}{\begin{array}{c} 100\%\mathrm{Absorbance}\mathrm{value}\mathrm{of}\mathrm{control}\mathrm{group}-\mathrm{Absorbance}\mathrm{value}\mathrm{of}\mathrm{blank}\mathrm{group} \end{array}}$$

#### Morphological observation

The differences in cell morphology and number between the different treatment groups were compared by fluorescence microscopy. RAW264.7 cells were inoculated in 6-well plates (5 × 10^5^ cells/mL) at 1 mL per well, and blank, VC (100 μg/mL), and control (12.5–200 μg/mL) were set up in the same way as the MTT assay, and after 24 h of incubation, respectively. Cells were observed and photographed under a 200 × microscope using a fluorescent microscope to compare differences in cell morphology and number between different EFP-2 concentrations.

#### Flow cytometric analysis of apoptosis

The 12-well plates were seeded with good cells plastered overnight (5 × 10^5^ cells/well), and VC, EFP-2 in concentration gradient, and 1.2 Mm H_2_O_2_ were added. Another sample was set as blank control and 1.2 mM H_2_O_2_ was used as the positive control, and the cells were incubated in the incubator for 4 h.

We transferred the collected cells to a 1.5 mL EP tube with PBS and divided it into two parts, one for the blank control group and the other part for the same volume as the sample tube, as the No stain group. Dilute 10 × Binding Buffer with PBS, dilute BV421 Annexin V with Binding Buffe: BV421 Annexin V = 50:1 volume ratio, add 70 μL per tube to the sample tube, and place on ice for 30 min away from light. The nucleic acid dye 7-AAD was added to 1 μL per tube and left for 10 min at room temperature and protected from light (BV421 Annexin V and 7-AAD can also be stained at the same time, considering that BV421 Annexin V’s staining ability is lower than that of 7-AAD, BV421 Annexin V is stained first). Add PBS 800 μL/tube, centrifuge, discard the supernatant, and add 200 μL PBS to make cell suspension. Filter on the machine and flow cytometry for testing.

#### Protective effects of EFP-2 in a zebrafish model of oxidative damage

Zebrafish parents are kept in the zebrafish breeding system, quality control reference “B/T 39649-2020 experimental animals Experimental fish quality control.” Ten pairs of parental fish, male and female, were temporarily reared in 3 L paired boxes, and fertilized eggs with basically synchronized development were obtained using the photoinduction method; 3 dpf embryos were selected as experimental organisms. The experiment was set up the control group (blank control group), the GSH group (positive drug control group, 100 μM) and the subject group (200 μg/L). The incubation was continued for 24 h after administration. The rest of the operation was performed according to the standard “Rapid assay for zebrafish model of antioxidant function of T/ZHCA health food (ROS method).” After the experiment, the live imaging of each group of pups was performed using a Nikon fluorescence microscope (Ci-S), and no less than 10 fish were imaged in each group; the image grayscale values were extracted using ImageJ software, and the ROS clearance rate was calculated.

### Statistical analysis

Data were expressed as the mean ± standard deviation (SD) of three measurements. Statistical analysis was performed by one-way analysis of variance (ANOVA) and *t*-test in SPSS software to assess the significance of differences, and *p* < 0.05 was considered significant.

## Results and discussion

### Elderberry polysaccharide isolation and purification

**FIGURE 2:**
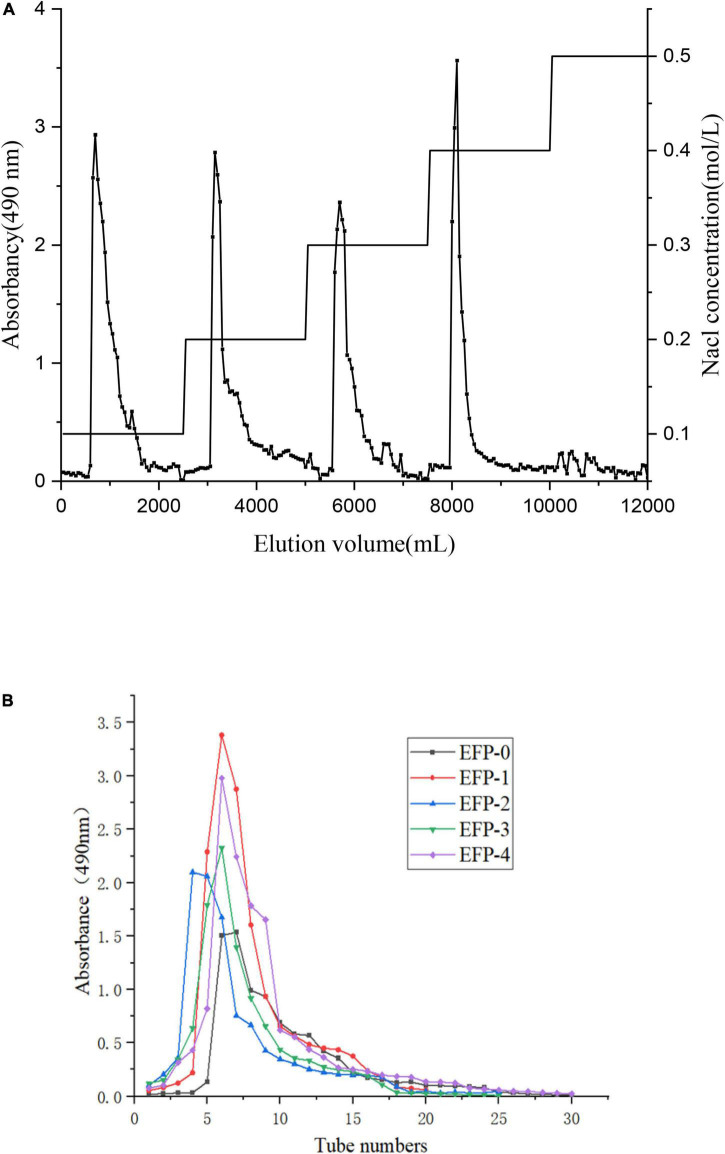
Isolation and purification of five polysaccharide fractions from elderberry fruits. **(A)** Elution profile of elderberry polysaccharides on a DEAE cellulose-52 column. **(B)** Elution profiles of five fractions (EFP-0, EFP-1, EFP-2, EFP-3, and EFP-4) on a Sephadex G-100 column.

The crude polysaccharide (EFP) was extracted from elderberry fruits in 2.2 ± 0.4%. Then five polysaccharides were isolated, two neutral polysaccharides (EFP-0 and EFP-1) and three acidic polysaccharides (EFP-2, EFP-3, and EFP-4), and were obtained by passing through a DEAE-52 cellulose ion-exchange column (Figure 2A), with recoveries of 6.74, 6.03, 5.83, 4.69, and 7.24%, respectively. Yuan et al. (43) reported that five polysaccharides were also isolated and purified from blackened jujube. These five polysaccharide fractions were then further purified on a Sephadex G-100 column, and the purified five polysaccharide fractions all showed single peaks (Figure 2B), and the purity of polysaccharides was 77.86 ± 0.63, 81.67 ± 0.43, 90.65 ± 0.57, 81.77 ± 0.83, and 82.25 ± 0.56%, respectively, measured by phenol-sulfuric acid method.

### Molecular weight analysis

The molecular weight of EFP was determined by HPGPC, and the molecular weight distribution of polysaccharides is critical for biological activity (44). The results were shown in Table 1, and based on the standard curve equation: lgMw = −0.1889 *t* + 12.007 (*R*^2^ = 0.9943), it was calculated that among the five polysaccharides, EFP-2 had the highest heavy average molecular weight of 7.7638 × 10^6^ g/mol, followed by EFP-1 (7.0523 × 10^6^ g/mol), EFP-4 (7.3173 × 10^5^ g/mol), and EFP-0 (1.7981 × 10^6^ g/mol) and the lowest heavy average molecular weight was EFP-3 (4.3855 × 10^5^ g/mol) with retention times of 27.088, 32.518, 27.309, 33.695, and 30.451 min. Among them, the polydispersity index (Mw/Mn) of EFP-0, EFP-1, and EFP-2 are 1.931, 2.132, and 2.147, respectively, close to 2, indicating that the molecular weight distribution of EFP-0, EFP-1, and EFP-2 were relatively narrow and the polymer structure was more homogeneous, so we speculate that EFP-0, EFP-1, and EFP-2 had better hydrophilicity and solubility (45).

**TABLE 1:** Molecular weight characteristic parameters.

Sample	RT (min)	Mw (g/mol)	Mn (g/mol)	Mp (g/mol)	Mw/Mn
EFP-0	30.451	1.7981 × 10^6^	9.3108 × 10^5^	1.8289 × 10^4^	1.931
EFP-1	27.309	7.0523 × 10^6^	3.3072 × 10^6^	1.2336 × 10^4^	2.132
EFP-2	27.088	7.7638 × 10^6^	3.6156 × 10^6^	1.7536 × 10^4^	2.147
EFP-3	33.695	4.3855 × 10^5^	2.5156 × 10^5^	2.2593 × 10^4^	1.743
EFP-4	32.518	7.3173 × 10^5^	4.0443 × 10^5^	5.0097 × 10^4^	1.809

### Analysis of monosaccharide composition

Monosaccharide composition analysis is a basic step in determining the structural characterization of polysaccharides, and Table 2 showed that the five polysaccharides are heteropolysaccharides, of which EFP-0 and EFP-1 do not contain galacturonic acid as neutral sugars, EFP-2, EFP-3, and EFP-4 all contain galacturonic acid as acidic sugars, and the proportion of galactoalic acid is 6.90%:22.70%:30.60%, respectively. EFP-0, EFP-1, EFP-2, EFP-3, and EFP-4 all contain Arabia (47.70%:55.00%:41.40%:19.20%:23.20%), Glucose (15.00%:16.30%:18.00%:15.40%:19.40%), Galactose (14.10%:22.30%:24.80%:10.10%:16.10%), Mannose (4.10%: 2.40%:6.60%:7.50%:8.70%), and glucosamine hydrochloride (1.50%:4.00%:2.30%:1.10%:2.00%), except that EFP-0 and EFP-3 both contain fucose (17.60%:24.10%). Notably, although rhamnose is widely found in plant polysaccharides and pectins, none of the five polysaccharide fractions contained rhamnose. At the same time, the five components contain mannose and glucose, indicating that the polysaccharides isolated and purified by EFP have antioxidant activity and are all polymer heteropolysaccharides (46). Ferreira et al. (3) concluded that elderberry contains glucose, fructose, and a small amount of sucrose. This was similar to the monosaccharide composition of straw mushroom polysaccharides derived by Tian et al. (47), as they are all mainly composed of arabinose, mannose, glucose, and galactose. Data were suggesting that the combinations and ratios of monosaccharides were different, as they were not always the same even in the same genus (48).

**TABLE 2:** Composition and percentage of monosaccharides.

Sample	Arabinose (%)	Galactose (%)	Glucose (%)	Mannose (%)	Fucose (%)	Galacturonic acid (%)	Glucosamine hydrochloride (%)
EFP-0	47.70	14.10	15.00	4.10	17.60	0.00	1.50
EFP-1	55.00	22.30	16.30	2.40	0.00	0.00	4.00
EFP-2	41.40	24.80	18.00	6.60	0.00	6.90	2.30
EFP-3	19.20	10.10	15.40	7.50	24.10	22.70	1.10
EFP-4	23.20	16.10	19.40	8.70	0.00	30.60	2.00

### Structural identification of elderberry polysaccharides

#### Fourier transform infrared spectroscopy

**FIGURE 3:**
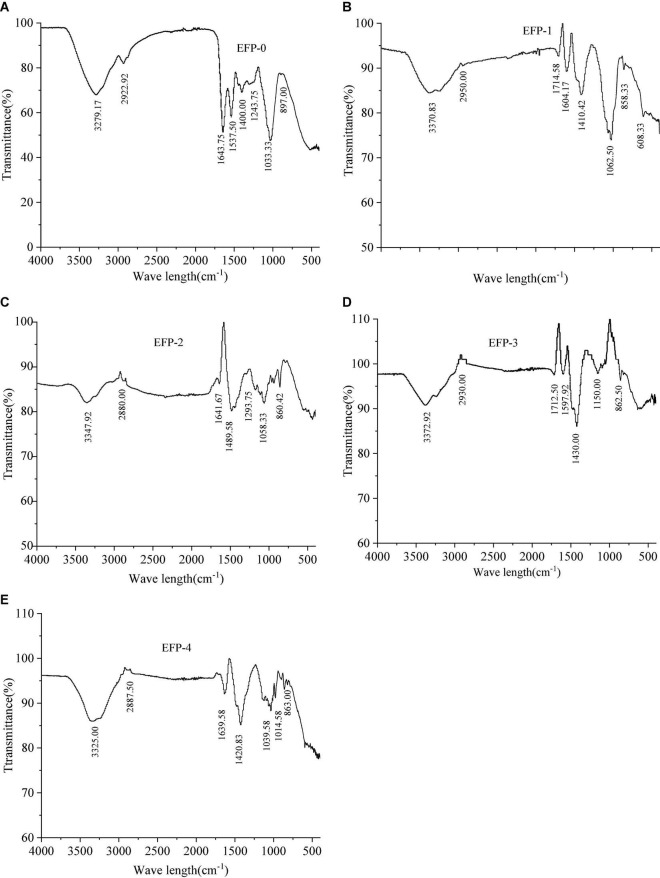
Infrared spectra of the five polysaccharide fractions of elderberry fruits and **(A–E)** represents the infrared spectra of EFP-0, EFP-1, EFP-2, EFP-3, and EFP-4, respectively.

Fourier transform infrared spectroscopy spectra revealed the main functional groups of polysaccharides. The FT-IR spectra as shown in Figure 3 indicated that the IR spectra of all five components contained similar absorption peaks in the range of 4,000 to 500 cm^–1^, only the absorption intensities differ. EFP-0, EFP-1, EFP-2, EFP-3, and EFP-4 can observe a broad and intense stretching peak in all FTIR spectrum around 3,200 to 3,400 cm^–1^, which was mainly caused by polysaccharide molecules or intermolecular O-H stretching vibrations, indicating the presence of intermolecular hydrogen bonds (49, 50). The weak absorption bands around 2,900 to 2,800 cm^–1^ (Figures 3A–E) were attributed to C-H, -CH_2_-, and -CH_3_- asymmetric stretching vibrations (51), and the presence of these two absorption peaks suggests that all five components purified from elderberry polysaccharides are carbohydrates, but EFP-0 is relatively strong (43). The absorption peak near 1,400 to 1,700 cm^–1^ was due to the -COOH bending vibration or symmetric stretching vibration of C-O, indicating the presence of glucuronic acid in EFP and the presence of -COOH groups; in particular, EFP-2 has the largest carboxyl peak (52–54). EFP-3 has an absorption peak at 1,597.92 cm^–1^, indicating the presence of N-H variable angle vibration in EFP-3 (49). The absorption peaks at 1,000 to 1,200 cm^–1^ were related to the C-O-C stretch of the glycoside bonds, which have different spectral shapes for polysaccharides composed of different monosaccharides, where multiple absorption bands appeared in EFP-2, EFP-3, and EFP-4, but only 1–2 absorption bands in EFP-0 and EFP-1 (Figures 3A,B); and it is noteworthy that EFP-0, EFP-2, EFP-3, and EFP-4 (Figures 3A,C–E) have absorption peaks at 1,000 to 1,050 cm^–1^, indicating the presence of the pyranose ring in these four polysaccharide fractions (55), and 1,024 cm^–1^ is the peak of C-O-C stretching vibration (56). A peak around 900 cm^–1^ indicates the presence of β-glycoside bonds in the polysaccharide chain, but the peaks of EFP-0 and EFP-1 are weaker, and there is an absorption peak around 850 cm^–1^ indicating the presence of α-glycoside bonds in polysaccharides, but only EFP-0 has no absorption peak here (45, 57, 58) and (59). Thus, the FTIR spectra of these purified polysaccharide components all have typical absorption peaks of heteropolysaccharides.

#### X-ray diffraction analysis

**FIGURE 4:**
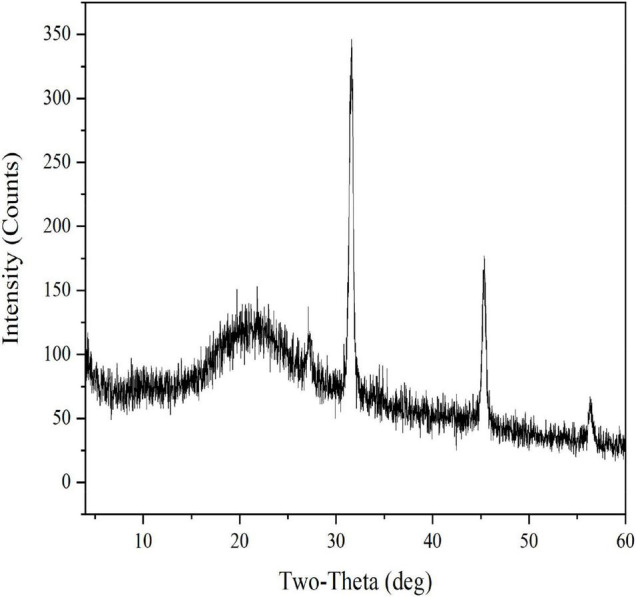
X-ray diffraction curve of EFP-2.

EFP-2 was determined by X-ray diffraction to be an amorphous or crystalline structure. Generally, polysaccharides with sharp narrow diffraction peaks are crystalline structures, while polysaccharides with broad diffraction peaks are amorphous structures (60). As shown in Figure 4, the XRD profile of EFP-2 has a strong broad peak at 2θ = 20°, and a similar peak pattern was observed in the 2θ region by Zhao et al. (61) and Ji et al. (50). A new sharp crystal peak at approximately 2θ = 32, 46, and 56° each indicates that EFP-2 had an ordered crystal conception, and similar results were observed by Yuan et al. (62) and Bai et al. (63), which may be related to the content of glyoxylate, an acidic polysaccharide containing three peaks with sharp and narrow diffraction (64). This inference was consistent with the results for monosaccharide composition.

#### Scanning electron microscope

**FIGURE 5:**
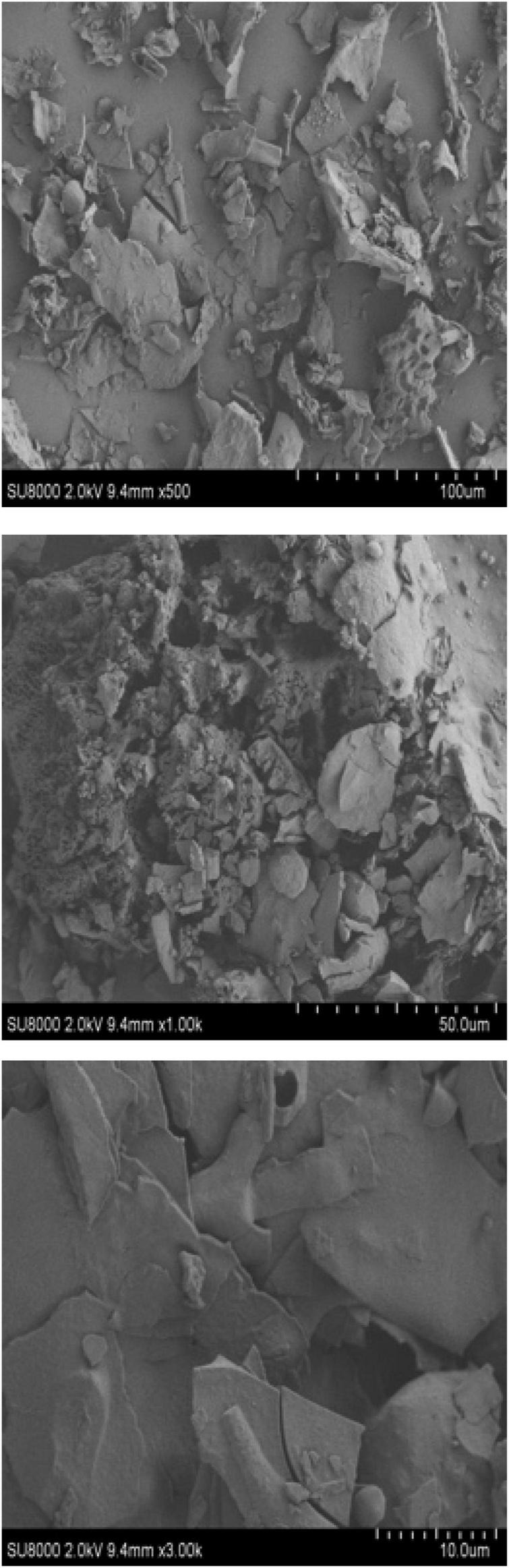
SEM photo of the surface structure of EFP-2.

Scanning electron microscopy can be used as a qualitative tool to analyze the surface morphology of polysaccharides (64). As can be seen from Figure 5, the polysaccharide structure appears fragmented at 500 × magnification and had a rough surface, which may be caused by various hydroxyl and carboxyl groups (64). At 1,000 × magnification, the EFP-2 polysaccharide surface showed agglomerates or lamellar aggregates, but no regularity overall, which may be due to the magnetic field enhancement leading to different degrees of cellular tissue damage, resulting in wrinkling, cracking, and rupture (21). At 3,000 × magnification, the surface of EFP-2 polysaccharide was a smooth sheet-like structure with tighter binding and surface distribution, possibly due to stronger interactions between polysaccharide molecules (65, 66).

#### Methylation analysis

Methylation analysis is the most widespread and effective method for determining the type and linkage of polysaccharide glycosidic bonds (44, 67). To analyze the linkage mode of monosaccharides in EFP-2, the polysaccharide structure was analyzed by methylation, the reaction products were hydrolyzed and acetylated sequentially, and the resulting final products were analyzed by GC-MS to obtain information on the linkage mode of monosaccharides.

**FIGURE 6:**
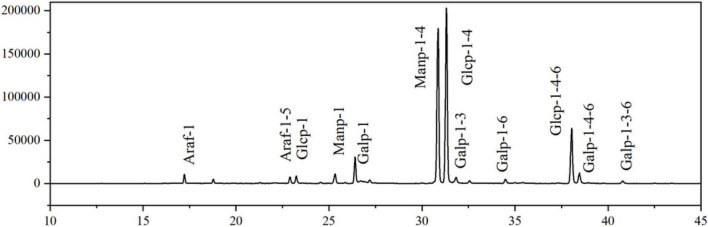
Total ion chromatogram of the methylated production of EFP-2.

Figure 6 showed that the total ion flow diagram of the methylation products and the analysis of the polysaccharide methylated glycol acetates results were listed in Table 3. It can be seen that EFP-2 included 12 glycosidic bond types, and 1,4-Glc*p* (34.90%), 1,4-Man*p* (32.70%), and 1,4,6 -Glc*p* (10.80%) were the three major glycosidic bond linkage mode, with small amounts of t-Gal*p* (5.10%), t-Man*p* (4.40%), t-Ara*f* (2.50%), t-Glc*p* (2.40%), 1,4,6-Gal*p* (1.90%), 1,5-Ara*f* (1.90%), 1,3-Gal*p* (1.30%), 1,6-Gal*p* (1.00%), 1,3,6-Gal*p* (1.00%), and 1,3,6-Gal*p* (0.50%) residues. It can be seen that the EFP-2 backbone may be joined by 1,4-α-Glc*p* and 1,4-α-Man*p*, and that 1,4-α-Glc*p* produced a branched chain at the C-6 position.

**TABLE 3:** Analysis of polysaccharide methylated glycol acetates (PMAA) results.

RT	Methylated fragments	Mass-to-charge ratio (m/z)	Molar ratio (%)	Glycosidic bond type
17.225	2,3,5-Me_3_-Ara*f*	43,71,87,101,117,129,145,161	2.50	Ara*f*-(1→
22.903	2,3-Me_2_-Ara*f*	43,71,87,99,101,117,129,161,189	1.90	→5)-Ara*f*-(1→
23.233	2,3,4,6-Me_4_-Glc*p*	43,71,87,101,117,129,145,161,205	2.40	Glc*p*-(1→
25.325	2,3,4,6-Me_4_-Man*p*	43,71,87,101,117,129,145,161,205	4.40	Man*p*-(1→
26.394	2,3,4,6-Me_4_-Gal*p*	43,71,87,101,117,129,145,161,205	5.10	Gal*p*-(1→
30.868	2,3,6-Me_3_-Man*p*	43,87,99,101,113,117,129,131,161,173,233	32.70	→4)-Man*p*-(1→
31.307	2,3,6-Me_3_-Glc*p*	43,87,99,101,113,117,129,131,161,173,233	34.90	→4)-Glc*p*-(1→
31.841	2,4,6-Me_3_-Gal*p*	43,87,99,101,117,129,161,173,233	1.30	→3)-Gal*p*-(1→
34.497	2,3,4-Me_3_-Gal*p*	43,87,99,101,117,129,161,189,233	1.00	→6)-Gal*p*-(1→
38.051	2,3-Me_2_-Glc*p*	43,71,85,87,99,101,117,127,159,161,201	10.80	→4,6)-Glc*p*-(1→
38.465	2,3-Me_2_-Gal*p*	43,71,85,87,99,101,117,127,159,161,201,261	1.90	→4,6)-Gal*p*-(1→
40.806	2,4-Me_2_-Gal*p*	43,87,117,129,159,189,233	0.50	→3,6)-Gal*p*-(1→

As can be seen in the methylation results, the number of glycosidic bonds differs from the composition of the monosaccharides measured, mainly due to the fact that methylation analysis is more qualitative than quantitative, and the percentage content of GC-MS is different due to the lower sensitivity of ion chromatography. In addition, EFP-2 has good solubility, and the use of DMSO reagents can avoid the problem of insufficient methylation of polysaccharides, so the glycosidc bond type determined by the methylation experiment is reliable (68).

#### NMR spectroscopy analysis of EFP-2

**FIGURE 7:**
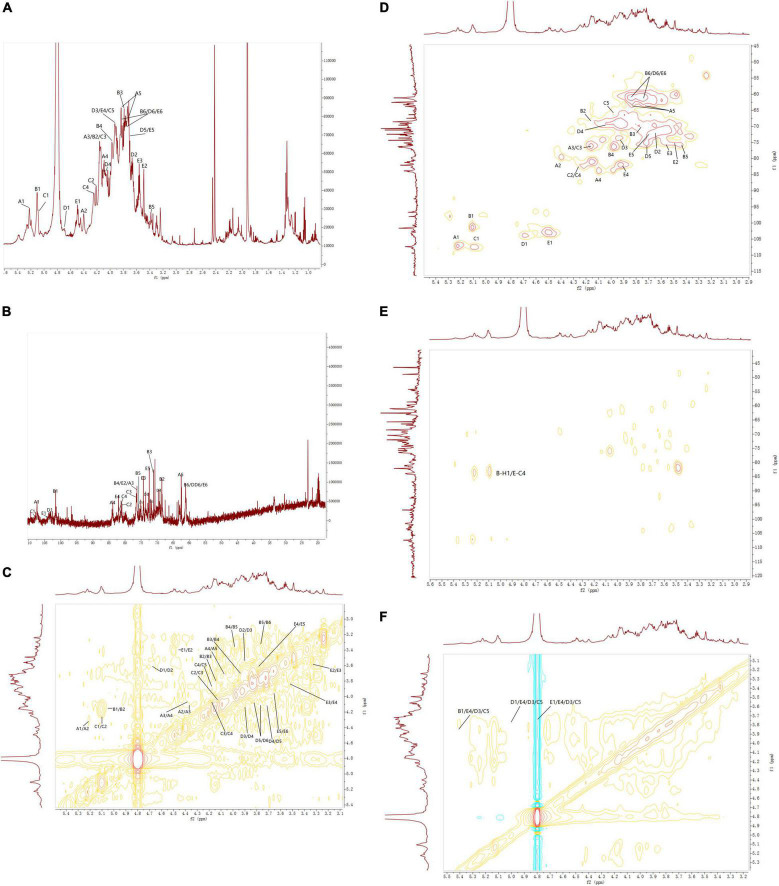
NMR spectra of EFP-3 in elderberry. **(A)** 1H NMR; **(B)** 13C NMR; **(C)** 1H-1H COSY; **(D)** HSQC; **(E)** 5-HMBC; and **(F)** NOESY.

According to the ^1^H NMR spectrum (Figure 7A), EFP-2 had five main anomeric proton signals at δ 5.22, 5.10, 5.09, 4.70, and 4.45, which were labeled A, B, C, D, and E, respectively. The chemical shifts from 3.21 to 4.44 ppm in the ^1^H NMR spectrum were assigned to protons from C-2 to C-6 in the residues. The corresponding anomeric carbon signals in the ^13^C NMR spectrum were labeled with reference to the HSQC data. All the ^1^H and ^13^C NMR (Figure 7B) of labeled residues were assigned with data from the ^1^H-^1^HCOSY (Figure 7C) and HSQC spectra (Figure 7D). In ^13^C NMR spectrum(Figure 7B), four isomers with heteropolycarbon resonance signals were at δ_*C*_ 90 to 110 ppm (107.30, 101.59, 107.48, 104.03, and 102.91 ppm). This indicated that EFP-2 contained both α and β configurations in its structure (69), which was consistent with the results of the FTIR spectrum. All the ^1^H and ^13^C signals were assigned as completely as possible according to 2D NMR analysis and literature values. The chemical shift in the anomeric proton of residue A was δ 5.22. The corresponding chemical shift in the anomeric carbon was δ 107.30. The other protons of residue A were assigned from the COSY spectrum. The other corresponding carbon and hydrogen signals were δ 79.65 (4.30), 76.38 (4.11), 84.03 (4.10), and 62.54 (3.82, 3.72), determined with HSQC (Figure 7D). Based on these NMR data, we inferred that T-α-L-Araf (1→ was a constituent unit of EFP-2 Combined with the analysis, we inferred that the →4) –Man*p* (1→ was assigned as residue B, →5) -α-L-Ara*f* (1→ as residue C, T-β-D-Gal*p* (1→ as residue D, and →4) -β-D- Glc*p* (1→ as residue E (71–73). Taking the results of methylation analysis into consideration, it was supposed that the presence of two different terminal signals was suggested as T-α-L-Ara*f* (1→ and T-β-D-Gal*p* (1→ in EFP-2, as well as comparing their NMR data with those in references (Table 4; 70). In the HMBC spectrum, some inter-residual cross-peaks were observed: B-H-1 to E-C-4 (Figure 7E). In the NOESY spectrum, some inter-residual cross-peaks were observed: B-1 to E-4/D-3 and C-5; E-1 to C-5/D-3 and E 4; D-1 to C-5/D-3 and E 4 (Figure 7F). The determination of the monosaccharide composition analysis confirmed that EFP-2 was mainly composed of galactose, arabinose, glucose, and mannose. Based on its monosaccharide composition and 1D and 2D NMR spectroscopy results, EFP-2 was proposed to comprise two units of →4) –Man*p* (1→4) -β-D-Glc*p* (1→ and →4) -β-D-Glc*p* (1→ 5) -α-L-Ara*f* (1→.

**TABLE 4:** Assignments of ^1^H and ^13^C NMR spectra for EFP-2.

			1	2	3	4	5	6
A	T-α-L-Ara*f* (1→	C	107.30	79.65	76.38	84.03	62.54	
		H	5.22	4.30	4.11	4.10	3.82/3.72	
B	→4)-Man*p* (1→	C	101.59	68.69	71.10	76.49	75.77	61.16
		H	5.10	4.24	3.76	3.98	3.46	3.86/3.76
C	→5)-α-L-Ara*f* (1→	C	107.48	81.90	76.56	82.03	66.35	
		H	5.09	4.23	4.15	4.25	3.90	
D	T-β-D-Gal*p* (1→	C	104.03	72.01	73.59	69.15	75.12	61.16
		H	4.70	3.58	3.84	4.02	3.72	3.86/3.76
E	→4)-β-D-Glc*p* (1→	C	102.91	76.27	74.31	82.10	72.43	61.16
		H	4.45	3.42	3.58	3.85	3.72	3.86/3.76

### Protective effect of H_2_O_2_-induced oxidative stress

#### Effect of EFP-2 on H_2_O_2_-induced cell competence in RAW264.7 cells

**FIGURE 8:**
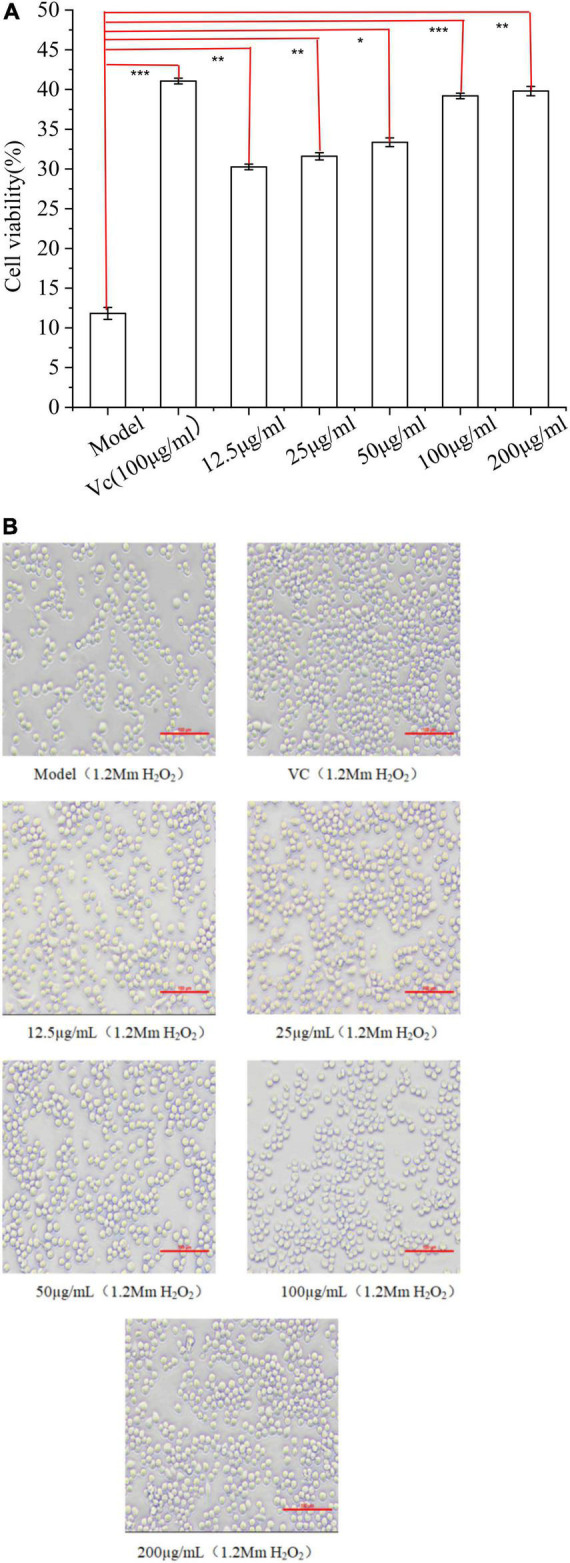
**(A)** Effect of EFP-2 on the survival rate of RAW264.7 cells with H_2_O_2_-induced injury; **(B)** comparison of the differences in morphology and number of RAW264.7 cells with H_2_O_2_-induced injury at different EFP-2 concentrations. Significant differences with model group were designated as *P < 0.05, **P < 0.01, ***P < 0.001.

Compared with the model group (Figure 8A), the survival rate of cells under 1.2 mM H_2_O_2_ conditions both increased with the increase of polysaccharide concentration, which acted as a protective anti-free radicals *in vitro*, indicating that EFP-2 had a strong protective effect on RAW264.7 cells with H_2_O_2_-induced damage. It was shown that cell viability was enhanced with increasing concentrations, suggesting a dose-dependent relationship between polysaccharide concentration and cellular activity (12.5–200 μg/mL), which was consistent with the studies reported by Wang et al. (74) and Xie et al. (75). In Figure 8B, the cells were mostly round, with smooth surfaces and uniform size in clusters (76). Cell density and cell viability increased significantly after 24 h treatment with EFP-2 (12.5–200 μg/mL), which became more pronounced with increasing EFP-2 concentration, suggesting that FFP-2 could inhibit morphological changes in RAW264.7 cells and protect them from H_2_O_2_-induced oxidative stress. This may be because the inhibitory effect of EFP-2 on cellular death induced by H_2_O_2_ oxidative stress is determined by monosaccharides, which can provide the ability of cells or control oxidative stress (77). Studies have shown that many plant polysaccharides can scavenge excess free radicals and play an antioxidant role, and Li et al. (78) have concluded that red mushroom polysaccharides also have a significant protective effect on the activity of H_2_O_2_-induced RAW264.7 cells.

#### Apoptosis analysis

**FIGURE 9:**
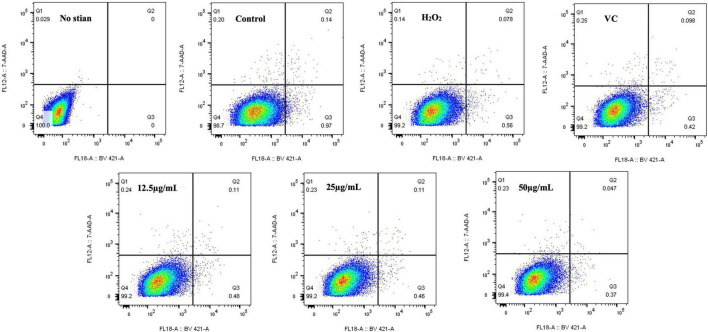
Effect of EFP-2 on H_2_O_2_-induced apoptosis in RAW264.7 cells.

Studies have shown that H_2_O_2_-induced oxidative stress can induce DNA damage, inflammation, tissue damage, and apoptosis, which is a key target of most chemotherapy drugs because it is the primary mechanism for clearing damaged cells (75). As shown in Figure 9, the percentage of apoptosis in the H_2_O_2_ group was 0.56%, the percentage of apoptosis in RAW264.7 cells treated with EFP-2 was significantly reduced to less than 0.50%, and the percentage of apoptosis in RAW264.7 cells gradually decreased to 0.48, 0.46, and 0.37% with the increase of EFP-2 concentration (12.5–50 μg/mL). Compared with VC, RAW264.7 cells treated with EFP-2 (<25 μg/mL) had a weaker apoptotic activity, which was enhanced when the concentration of EFP-2 (50 μg/mL) was increased to a certain concentration. These results suggested that EFP-2 can participate in the process of programmed suicide to remove non-functional, unwanted, abnormal, and harmful cells, reducing the death of RAW264.7 cells. Thus, EFP-2 was shown to protect cells from H_2_O_2_-induced oxidative stress.

#### Protective effect of EFP-2 polysaccharide on oxidative damage model in zebrafish

**FIGURE 10:**
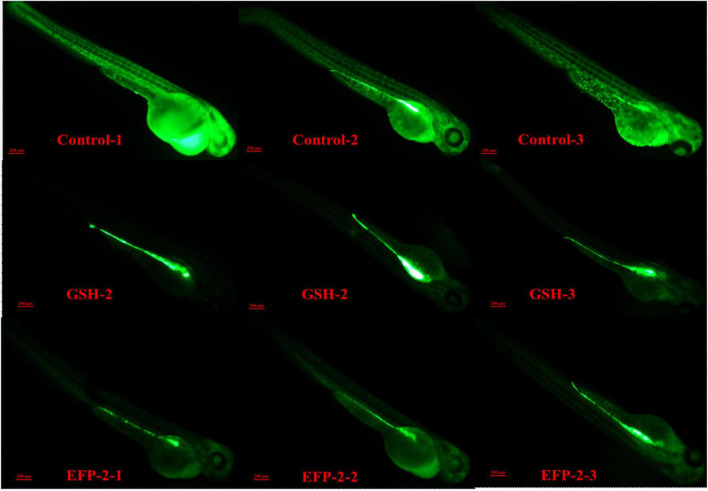
Partial fluorescence map in zebrafish.

Zebrafish are often used as biological models for *in vivo* studies of small molecules and drugs because of their fast growth rate, small size, transparency, and ease of handling (77, 78). Glutathione, as a key antioxidant element, with the formation of its disulfide dimer, can respond to chemical stress and clear ROS, thereby balancing the intracellular redox homeostasis, helping to prevent oxidative stress in cells, and maintain a normal immune system (74, 79). The intensity of green fluorescence is positively correlated with the level of ROS, and the higher the brightness, the higher the level of ROS. When the level of ROS increases in the body, it will consume the antioxidant substances in the body and disrupt the homeostasis of the antioxidant defense system in the body. If the synthesis rate of antioxidant substances in the body is slow or the clearance rate of ROS is not fast, it will cause the peroxidation level of lipids and proteins in the body to increase, leading to the occurrence of chronic diseases and causing aging and other physiological phenomena related to peroxidation in the body (22, 80). Compared with the blank control group (Control group), both the positive drug control group (GSH group) and the subject group (EFP-2 group) could significantly reduce the overall ROS level of zebrafish litter, and the ROS clearance rate was 50.51 and 44.62% (Table 5), respectively. After the addition of EFP-2, the fluorescence intensity of zebrafish was significantly reduced (Figure 10), and the ROS level in zebrafish was decreased, which improved its antioxidant level, directly reflecting that EFP-2 has an antioxidant ability. In addition to the direct antioxidant response, polysaccharides may also activate some signaling pathways that protect zebrafish from oxidative stress damage. For example, Yang et al. (40) added HJP-1a at doses of 5, 25, and 50 μg/mL, which can increase the fluorescent spots of zebrafish embryos by 60.8 to 83.3%, and then achieve the best effect on the protective effect of oxidative damage cells.

**TABLE 5:** Fluorescence grayscale values for each group.

Serial number	Blank group	GSH group	Subjects group
1	434309	290555	205835
2	442634	195570	273728
3	325941	156175	155579
4	337943	215241	226494
5	344125	142899	243585
6	474124	242847	165656
7	433179	197831	209262
8	482588	199821	283857
9	460675	257933	251640
10	553025	149162	231248
11	302460	174076	239522
12	307248	/	/
Average value	408188 ± 81619.53*^b^*	202010 ± 46949.15*^a^*	226037 ± 40112.29*^a^*

Values with different superscripts indicate significant differences (P < 0.05).

## Conclusion

Five polysaccharide fractions, including two neutral polysaccharide fractions (EFP-0 and EFP-1) and three acidic polysaccharide fractions (EFP-2, EFP-3, and EFP-4), were isolated and purified from elderberry fruits using DEAE-52 anion-exchange chromatography and Sephadex G-100 dextran gel chromatography columns, and they differed in terms of monosaccharide composition and molecular weight. Both EFP-0 and EFP-1 were mainly composed of arabinose, galactose, and glucose, and fucose was also a major component of EFP-0. EFP-2, EFP-3, and EFP-4 were all acidic heteropolysaccharides, all containing arabinose (41.10, 19.20, 23.20%), galactose (24.80, 10.10, 16.10%), glucose (18.00, 15.40, 19.40%), and galacturonic acid (6.90, 22.70, 30.60%). The molecular weights were 1.7981 × 10^6^, 7.0523 × 10^6^, 7.7638 × 10^6^, 4.3855 × 10^5^, and 7.3173 × 10^5^ g/mol, respectively. The backbone of EFP-2 consisted of two units, →4) –Man*p* (1→4) -β-D-Glc*p* (1→ and →4) -β-D-Glc*p* (1→5) -α-L-Ara*f* (1→, and T-α-L-Ara*f* (1→ and T-β-D-Gal*p* (1→ residues were detected by methylation analysis and NMR analysis. In addition, the MTT method and zebrafish oxidative damage assay showed that EFP-2 had a protective effect on H_2_O_2_-damaged RAW264.7 cells in a dose-dependent manner, and zebrafish with the addition of EFP-2 would have low levels of ROS *in vivo* and also showed significant antioxidant activity. Therefore, the elderberry polysaccharide has antioxidant activity, which provides a theoretical basis for further studying the relationship between EFP-2 structure and antioxidant activity, and contributes to the development of EFP-2 as the most novel antioxidant. However, the disadvantage of this study is the lack of in-depth understanding of the relationship between polysaccharide structure and antioxidant activity.

## Data availability statement

The original contributions presented in this study are included in the article/supplementary material, further inquiries can be directed to the corresponding authors.

## Author contributions

XW, RZ, and JY contributed to the conception, design, and writing of this study. DW and JY contributed funding and materials for this study. FW contributed to the data organization of the manuscript. All authors contributed to this article and approved the submitted version.

## Conflict of interest

The authors declare that the research was conducted in the absence of any commercial or financial relationships that could be construed as a potential conflict of interest.

## Publisher’s note

All claims expressed in this article are solely those of the authors and do not necessarily represent those of their affiliated organizations, or those of the publisher, the editors and the reviewers. Any product that may be evaluated in this article, or claim that may be made by its manufacturer, is not guaranteed or endorsed by the publisher.
